# Interstitial microduplication at 2p11.2 in a patient with syndromic intellectual disability: 30-year follow-up

**DOI:** 10.1186/1755-8166-7-52

**Published:** 2014-08-19

**Authors:** Kyung Ran Jun, Reinhard Ullmann, Saadullah Khan, Lawrence C Layman, Hyung-Goo Kim

**Affiliations:** 1 Department of Laboratory Medicine, Inje University Haeundae Paik Hospital, Busan, South Korea; 2 Department of Human Molecular Genetics, Max Planck Institute for Molecular Genetics, Berlin, Germany; 3 Department of Biotechnology & Genetic engineering, Kohat University of Science & Technology (KUST), Kohat, Khyber Pakhtunkhwa, Pakistan; 4 Section of Reproductive Endocrinology, Infertility & Genetics, Department of Obstetrics and Gynecology, Institute of Molecular Medicine and Genetics, Medical College of Georgia, Georgia Regents University, 1120 15th Street, Augusta, Georgia; 5 Neuroscience Program, Medical College of Georgia, Georgia Regents University, Augusta, Georgia

**Keywords:** Array CGH, *CAPG*, Copy number variation, Duplication, Intellectual disability, Recurrent infection, *RNF181*, 2p11.2, *VAMP8*

## Abstract

**Background:**

Copy number variations at 2p11.2 have been rare and to our knowledge, no abnormal phenotype with an interstitial 2p11.2 duplication has yet been reported. Here we report the first case with syndromic intellectual disability associated with microduplication at 2p11.2.

**Results:**

We revisited a white female subject with a chromosome translocation, t(8;10)(p23;q23)mat and a 10q telomeric deletion suspected by G-banding 30 years ago. This female with severe intellectual disability, no speech, facial dysmorphism, intractable epilepsy, recurrent infection, and skeletal abnormalities has been observed from the birth until her death. The karyotype analysis reconfirmed the previously reported chromosome translocation with a revision as 46,XX,t(8;10)(p23.3;q23.2)mat by adding more detail in chromosomal sub-bands. The array comparative genomic hybridization, however, did not detect the 10q terminal deletion originally reported, but instead, revealed a 390 kb duplication at 2p11.2; 46,XX,t(8;10)(p23.3;q23.2)mat.arr[hg 19] 2p11.2(85469151x2,85474356-85864257x3,85868355x2). This duplication region was confirmed by real-time quantitative PCR and real-time reverse transcriptase quantitative PCR.

**Conclusions:**

We suggest three positional candidate genes for intellectual disability and recurrent infection based upon gene function and data from real-time reverse transcriptase quantitative PCR—*VAMP8* and *RNF181* for intellectual disability and *CAPG* for recurrent infection.

## Background

A variety of terminal 2p duplications known as partial 2p trisomy are the unbalanced chromosomal rearrangements resulting from malsegregation of various balanced translocations [1-3]. Interpretation of the clinical phenotypes of carriers with these unbalanced translocations is complicated due to the co-existence of terminal monosomy and terminal trisomy. Different isolated duplications within the 2p region had been reported in more than 20 patients, but they vary in size and location, ranging from 2p12 to 2p25 [4-7]. These duplications are associated with a syndromic phenotype including intellectual disability, growth and psychomotor retardation, delayed bone age, congenital heart abnormalities, pulmonary hypoplasia, hypoplastic kidney, genital anomaly, anencephaly, neural tube defects, finger and toe abnormalities, or dysmorphic facial features encompassing hypertelorism, prominent forehead, broad nasal bridge, low-set ear, and micrognathia [4-7]. Because the size and location of these 2p duplications may vary from individual to individual, comparison of overlapping regions for defining a minimal candidate region associated with a particular phenotype will be a useful strategy for identifying the causative gene as exemplified in the mapping of deletions [8,9].

Due to the limited resolution of banding and staining patterns of chromosomes in the pre-FISH (FISH-fluorescent *in situ* hybridization) and pre-CGH (CGH-comparative genomic hybridization) era, it is not uncommon to find misinterpretations of cytogenetic aberrations in the reported cases, which complicated attempts to assign a unique phenotype to a specific chromosome band. Here we revisited a case reported 30 years ago [10] and failed to confirm an ostensible 10q deletion. Instead, we identified a cryptic 2p11.2 microduplication by a microarray in the affected female subject. Some of her clinical features were developed years later in her life. To our knowledge, this is the first case with syndromic intellectual disability associated with an interstitial 2p11.2 duplication. Genotype/phenotype relationships have been discussed.

## Results

### Clinical report in a 30 year time period

The patient is a 30 year old white female with severe intellectual disability, a severe static encephalopathy, medically intractable epilepsy, and facial dysmorphism. She was first evaluated at the age of 10 months because of gastroenteritis and middle ear infection [10]. At that time, her height, weight and head circumference were in the 3rd percentile for chronological age. She is the first living child of healthy non-consanguineous marriage. The father was 42 years old and the mother with a previous spontaneous abortion was 26 years old at the birth of their daughter. Prenatal screening demonstrated normal alpha-fetoprotein levels for gestational age and a normal ultrasound, but studies indicated an Rh-sensitized status. The subject was a 2,700 g female at 35 weeks of gestation born by cesarean section.

**Figure 1:**
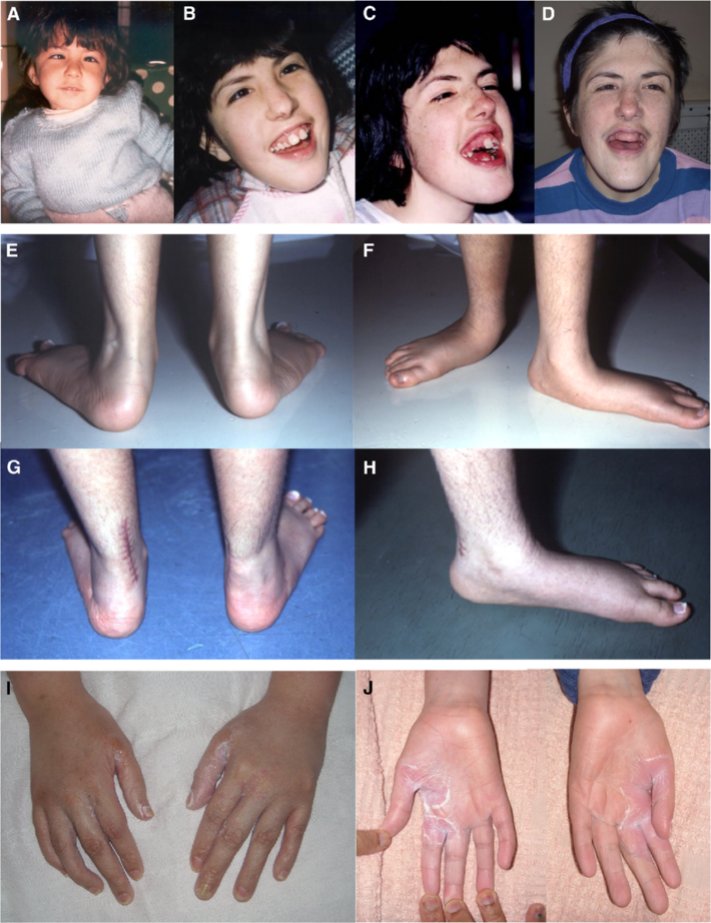
**Facial phenotype of the patient.** The frontal view of patient was shown chronologically at the age of 3 years showing strabismus **(A)**, at the age of 12 years after the surgery to correct strabismus. She had normal teething before taking anticonvulsant **(B)**, at the age of 18 years showing the side effects of damaged teeth and thickened gum from antiepileptic drugs **(C)**, and at the age of 30 years after the exodontias surgery of the rest of decayed teeth with facial dysmorphism characterized by long and thin face with prognathia and high nasal bridge **(D)**. She also had severe flaccid flatfeet **(E, F)**, which were successfully corrected by an orthopedic surgery lengthening the calcaneus with a homologuous graft from the iliac crest at the age of 16 years **(G, H)**. At the age of 31 years, she had fungal infection on the skin of her both hands that provoked a severe dermatitis **(I, J)**.

**Figure 2:**
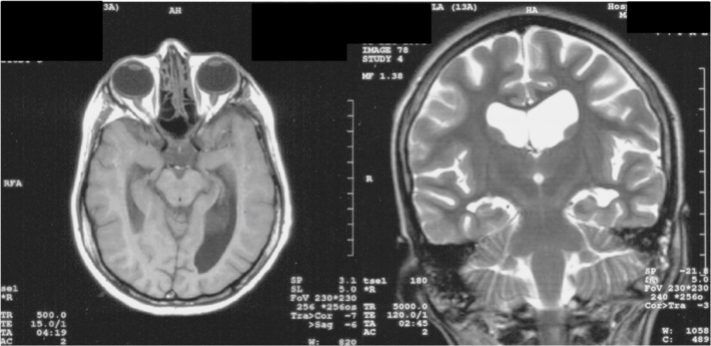
**The brain MRI views without contrast at the age of 14 years.** They showed cortical dysplasia and asymmetric lateral ventricles with an enlarged right ventricle.

After at the age of one year, she was able to sit and look into the eyes of anyone who spoke to her with responding smiles. However, the direction of her gaze was difficult to determine because of her convergent strabismus. She did not show any autistic behavior as evidenced by her continual demands on her mother. At the age of two years, she started to babble words such as mama, water, etc. Her facial appearances from 3 years to 30 years of age are shown chronologically in Figures 1A-D. When she was evaluated at 10 months of age, her phenotype was as follows: hypotonia, long and narrow palpebral fissures, long eyelashes, prominent eyebrow, mild hypertelorism, broad nasal bridge, long philtrum, short neck, the trunk and extremities of normal proportion with hirsutism, redundant subcutaneous tissue on arms, legs, and the dorsum of hands and feet, bilateral simian crease, infantile external genitalia, and convergent strabismus [10]. She began walking at 5 years of age.At 9 years, she was diagnosed with epilepsy, reporting myoclonic seizures with atypical absences. The electroencephalography (EEG) showed generalized 2 Hz spike-and-slow waves. At this time she lost her ability to verbalize simple words. She was started on valproic acid and then vigavatrin was added. Her EEG then showed anterior right temporal epileptiform discharges. Because of valproic acid side effects including hypothyroidism, teeth, and gum damage, this medication was replaced by clonazepam. Despite treatment, she continued to have seizures. She frequently had complex partial seizures with a moderate response to lamotrigine and oxcarbazepine. Olanzapine was also administered because of psychomotor excitation with episodes of autoaggression. Brain MRI without contrast revealed cortical dysplasia and asymmetric lateral ventricles with an enlarged right ventricle at the age of 14 years (Figure 2). Corrective surgery for her strabismus was performed at 12 years of age. In addition, orthopedic surgery for her severe flaccid flatfeet was also successfully performed by lengthening of the calcaneus with a homologous graft from her iliac crest at 16 years of age (Figures 1E-H).In her latest evaluation, the 30-year-old subject still suffers from intractable epilepsy. She has had growth, but she is below the third percentile. She has a severe mental and psychomotor delay with independent gait, but still no language. Additionally, she has global muscular atrophy, hypotonia, joint hyperflexibility (except for her elbows and knees which were rigid), scoliosis, and facial dysmorphism characterized by a long thin face with prognathia and high nasal bridge (Figure 1D). The mouth is usually open with sialorrhea, a full lower lip and a thin upper lip, showing thickened gums (Figure 1C), possibly related to antiepileptic medications. Several teeth are missing due to the first exodontia surgery at age 22 and the subsequent exodontia surgery of her remaining teeth, which showed decay at the age of 30. Her maxillofacial malformation worsened during adolescence with repeated changes of antiepileptic medications, which also caused inflammatory gum processes, hypersalivation, tooth loss, dental caries, and retraction of bone.The proband also has a low hairline, backward rotation of the ears, and small hands with hypoplastic fingernails and dry palmar and plantar surfaces. In addition, she has a fungal infection on the skin of her hands exclusively that has provoked a severe dermatitis (Figures 1I and J). This infection has been recurrent throughout her life. A recent brain MRI performed at the age of 29 did not confirm the previous diagnosis of cortical dysplasia, suggesting natural healing of the affected brain area. She died at the age of 31 years and 8 months under a grand-mal type of convulsion which took place during her bath and could not be controlled.

### The detection of microduplication at 2p11.2

**Figure 3:**
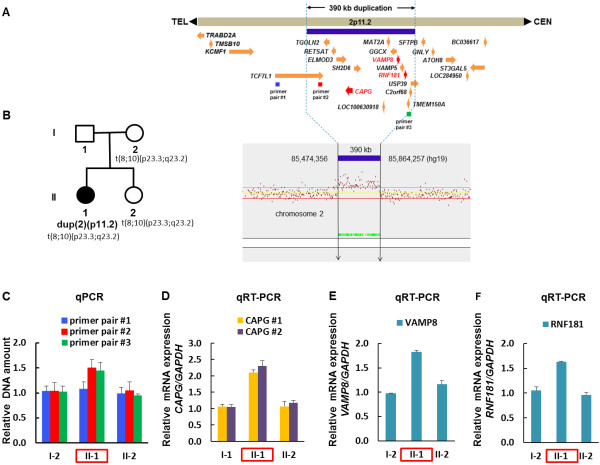
**Three candidate genes showing overexpression in qRT-PCR among 15 genes within the 2p11.2 duplication identified by array CGH and confirmed by qPCR. (A)** Array CGH showing the internal boundaries of minimal 390 kb heterozygous interstitial duplication at 2p11.2 showing 15 annotated genes involved. Three positional candidate genes, *CAPG* with recurrent infection and *VAMP8* as well as *RNF181* with intellectual disability, were depicted in red. Vertical arrows show duplicated region in array CGH and transcription direction is indicated by an arrow for each gene. **(B)** Pedigree of the family. Patient (II-1) with a multisystem developmental disorder and 2p11.2 microduplication shared the chromosome translocation with her asymptomatic mother (I-2) and younger sister (II-2). **(C)** The qPCR with genomic DNAs of the patient (II-1), her healthy sisier (II-2), and healthy mother (I-1) showed the duplication at 2p11.2 in the patient only from the primer pairs #2 and #3 of the duplicated region. **(D, E, F)** qRT-PCR showing the overexpression of three genes. The mRNA expression of each gene was increased by 1.7-2.1 folds in the patient compared to the unaffected mother and the sister.

Prenatal cytogenetic analysis using amniotic fluid showed a female fetus with an inherited chromosome translocation, t(8;10)(p23;q23) shared with her mother. The father’s karyotype was normal. When she was evaluated for developmental delay, her karyotype was reported as monosomy 10q, with a deletion from 10q23 to 10qter [10]. However, FISH using probes in this region did not confirm this deletion (data not shown). Another high resolution karyotype performed on peripheral blood lymphocytes using standard methods and G-banding techniques confirmed that the patient has a chromosome translocation, 46,XX,t(8;10)(p23.3;q23.2)mat, shared with her asymptomatic mother and younger sister. Methylation studies of the 15q11-q13 region demonstrated the presence of two fragments with sizes of 6.6 kb and 3.4 kb, indicating normal methylation status. Therefore a deletion or uniparental disomy in the critical imprinted region of Prader-Will and Angelman syndromes was highly unlikely (data not shown) However, array CGH analysis revealed a heterozygous 390 kb interstitial duplication (Chr2: 85,474,356-85,864,257 [hg 19]) in band 2p11.2 with a maximum size of 399 kb (Chr2: 85,469,151-85,868,355 [hg 19]) (Figure 3A).

Based on the aberrant karyotype and array CGH result found in the patient, the nomenclature is revised as 46,XX,t(8;10)(p23.3;q23.2)mat.arr[hg 19] 2p11.2(85469151x2,85474356-85864257x3,85868355x2).

### The confirmation of duplicated region by real-time quantitative PCR

**Figure 4:**
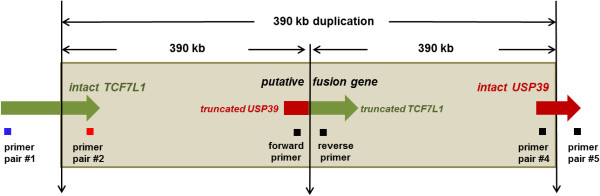
**Prediction of a putative fusion gene *USP39/TCF7L1 *due to the duplication.** If the distal and proximal duplication breakpoints truncate two genes, *TCF7L1* and *USP39*, it will produce a putative fusion gene *USP39/TCF7L1*, because the transcription direction of both genes is same. In order to amplify the putative fusion gene, a forward primer Fusion-USP39-F and a reverse primer Fusion-TCF7L1-R were used (Table 1).

The duplicated region was detected in the patient only and confirmed by real-time quantitative PCR (qPCR) (Figure 3C). The mRNA expression levels of *TCF7L1* and *USP39* were not different among patient (II-1), her mother (I-2), or her sister (II-2) by qRT-PCR using two primer pairs from each gene (Table 1, data not shown). The mRNA expression levels of *CAPG*, *VAMP8*, and *RNF181* were doubled in the duplication patient compared to her two normal sister and mother by qRT-PCR using two primer pairs designed within *CAPG* and one primer pair from *VAMP8* and *RNF181*, respectively (Figures 3D-F, Table 1). No amplicon of the putative fusion gene *USP39/TCF7L1* was obtained from RT-PCR, suggesting it is not expressed (Figure 4, Table 1).

**Table 1:** Primers used for qPCR, qRT-PCR, and RT-PCR

**Primer name**	**Gene name (GenBank accession number)**	**exon number**	**Primer sequence (5′ → 3′)**	*Tm (°C)
**qPCR**				
primer pair #1F	*TCF7L1*	exon1	F: ACGAGCTGATCCCCTTCC	60.16
primer pair #1R	(NM_031283)		R: CTGCTCTGGTTCTCCGACTC	60.14
primer pair #2F	*TCF7L1*	exon12	F: GCAAGAAGCCATGTGTTCAG	59.45
primer pair #2R	(NM_031283)		R: GGTTTCTGGTTTGGTGGTGA	60.79
primer pair #3F	*TMEM150A*	exon8	F: TACGAGTTTGGGGCAGTCTC	60.25
primer pair #3R	(NM_001031738)		R: CCTCCCCAGACCTTAGATCA	59.79
GAPDH-F	*GAPDH*	exon6	F: GATCATCAGCAATGCCTCCT	60.4
GAPDH-R	(NM_001289745)		R: ATGGCATGGACTGTGGTCAT	60.4
**qRT-RCR**				
primer pair #1F	*TCF7L1*	exon1	F: ACGAGCTGATCCCCTTCC	60.16
primer pair #1R	(NM_031283)		R: CTGCTCTGGTTCTCCGACTC	60.14
primer pair #2F	*TCF7L1*	exon12	F: GCAAGAAGCCATGTGTTCAG	59.45
primer pair #2R	(NM_031283)		R: GGTTTCTGGTTTGGTGGTGA	60.79
primer pair #4F	*USP39*	exon5	F: ATGTTCCTCCTCTCCGGAAC	60.46
primer pair #4R	(NM_006590)		R: AGCATCTCATGGGGAGACAC	60.08
primer pair #5F	*USP39*	exon10	F: GACCTCATTGCCAACATCGT	60.23
primer pair #5R	(NM_006590)	exon11	R: GTGTGATCATCTGGGGAAGG	60.33
CAPG-1F	*CAPG*	exon3	F: CTCCATTCCCAGGCTCAGT	60.21
CAPG-1R	(NM_001747)		R: GAAACCTCTTCTGGGCCATT	60.44
CAPG-2F	*CAPG*	exon9	F: CGAAAAGCGAATGAGAAGGA	60.46
CAPG-2R	(NM_001747)	exon10	R: CCACCCTCATTTCCAGTCC	60.31
VAMP8-F	*VAMP8*	exon 3	F: GAGGAAGCCAGTGAAGGTGG	60.6
VAMP8-R	(NM_003761)		R: CAGATCCTCTGTCTTGTTGCG	59.44
RNF181-F	*RNF181*	exon1	F: ACCAACATGCTGCTGGAGC	60
RNF181-R	(NM_016494)		R: TCTCAACCACAGTCTTGGCAG	59
GAPDH-F	*GAPDH*	exon6	F: GATCATCAGCAATGCCTCCT	60.4
GAPDH-R	(NM_001289745)		R: ATGGCATGGACTGTGGTCAT	60.4
**RT-PCR**				
Fusion-USP39-F	*USP39*	exon6	F: ATGTTCCTCCTCTCCGGAAC	60.46
Fusion-TCF7L1-R	TCF7L1 (*USP39/TCF7L1*)	exon5	R: TGTCTTTGGATCGATCTCTGG	60.20

*Tm: melting temperature.

## Discussion

It may seem surprising that the reinvestigation of this chromosome translocation, 46,XX,t(8;10)(p23.2;q23.2)mat has spanned a 30 year time period. Since this chromosome translocation was inherited from the healthy mother to the affected and unaffected daughters (Figure 3B), a pathological role of this translocation in the phenotype of the affected daughter was excluded. A previously reported 10q23-qter deletion was ruled out by FISH and array CGH, the latter of which instead identified a cryptic 390 kb duplication at 2p11.2 encompassing 15 genes (Figure 3A, Table 2). This genomic duplication was confirmed by qPCR (Figure 3C), suggesting its pathogenic role as a cryptic chromosome rearrangement not readily detected by conventional karyotype analysis done in 1983 [10]. The *de novo* status of this duplication is likely, but could not be confirmed because her father is unwilling to participate.

**Table 2:** Genes located at the duplicated region of 2p11.2

**Gene symbol**	**Gene name**	**Encoded protein**	**Function**	**Related disease**	**Remark**
*TCF7L1*	Transcription factor 7-like 1 (T-cell specific, HMG-box)	Transcription factor 7-like 1 (a member of T cell factor/lymphoid enhancer factor family of transcription factors)	Mediation of Wnt signaling pathway, regulation of cell cycle genes and cellular senescence	None	Homozygous mutant mice exhibit severe embryological defects particularly affecting the cardiovascular system, nervous system, and digestive system.
					Not affected by our duplication based on qRT-PCR result.
*TGOLN2*	Trans-golgi network protein 2	Trans-Golgi network integral membrane protein 2 precursor	Exocytic vesicle formation	None	No knock-out mice. A 7.8 kb deletion involving all 4 exons within the gene was reported in a normal control person.
*RETSAT*	Retinol saturase	All-trans-retinol 13,14-reductase precursor	All-trans-retinol 13,14-reductase activity, oxidoreductase activity	None	No knock-out mice.
*ELMOD3*	ELMO/CED-12 domain containing 3	ELMO domain-containing protein 3, isoform a, b,	Phagocytosis and cell migration	Deafness, autosomal recessive 88 (OMIM 615429)	No knock-out mice.
*CAPG*	Capping protein (actin filament), gelsolin-like	Macrophage-capping protein	Control of actin-based motility in non-muscle cells	None	Inactivation of this loci results in impaired immune cell motility which manifests in homozygous mutant mice as increased susceptibility to some bacterial infections.
*SH2D6*	SH2 domain containing 6	SH2 domain-containing protein 6	Unknown	None	No knock-out mice.
*LOC100630918*	Uncharacterized non-coding RNA	non-coding RNA	Unknown	None	No knock-out mice.
*MAT2A*	Methionine adenosyltransferase II, alpha	S-adenosylmethionine synthase	Production of S-adenosylmethionine from methionine and ATP	None	No knock-out mice. A 28 kb duplication containing this gene and GGCX was reported in a normal control person.
*GGCX*	Gamma-glutamyl carboxylase	Vitamin K-dependent gamma-carboxylase	Posttranslational modification of vitamin K-dependent protein	Autosomal recessive pseudoxanthoma elasticum-like disorder with multiple coagulation factor deficiency (OMIM 610842)	Only 50% of expected Ggcx(-/-) mice survive to term but the latter animals die uniformly at birth of massive intra-abdominal hemorrhage. A 28 kb duplication containing this gene and TAT2A was reported in a normal control person.
				Autosomal recessive vitamin K-dependent coagulation defect (OMIM 277450)	
*VAMP8*	Vesicle-associated membrane protein 8	Vesicle-associated membrane protein 8	Fusion of synaptic vesicles with the presynaptic membrane	None	Homozygous knock-out mice exhibit background-sensitive postnatal lethality, hydronephrosis, and reduced amylase secretion, type I hypersensitivity reaction, and platelet activation.
*VAMP5*	Vesicle-associated membrane protein 5	Vesicle-associated membrane protein 5	Docking and/or fusion of vesicles and cell membranes	None	No knock-out mice.
*RNF181*	Ring finger protein 181	E3 ubiquitin-protein ligase RNF181	E3 ubiquitin ligase activity	None	No knock-out mice. A 36 kb deletion encompassing the whole gene was reported in a normal control person.
*TMEM150A*	Transmembrane protein 150A	Transmembrane protein 150A precursor	Unknown	None	No knock-out mice.
*C2orf68*	Chromosome 2 open reading frame 68	UPF0561 protein C2orf68	Unknown	None	No knock-out mice.
*USP39*	Ubiquitin specific peptidase 39	U4/U6.U5 tri-snRNP-associated protein 2	Ubiquitin thiolesterase activity, zinc ion binding	None	No knock-out mice.
					A 12 kb deletion encompassing exons 4–6 and a 5.6 kb deletion encompassing exons 7–9 was reported in normal control persons.
					Not affected by our duplication based on qRT-PCR result.

Among the genes duplicated, the following CNVs have been identified in healthy control persons, thereby potentially ruling out them as candidate genes if gene dosage effect is underlying mechanism: a 7.8 kb deletion involving all 4 exons within the gene *TGOLN2*[11], a 28 kb duplication containing two genes, *MAT2A* and *GGCX*[12], a 36 kb deletion encompassing whole gene *RNF181*[13], and a 12 kb deletion encompassing exons 4–6 [14] and a 5.6 kb deletion encompassing exons 7–9 [15] of the gene *USP39* (Table 2).

Interstitial deletions encompassing 2p11.2 are particularly rare, with only seven cases reported to date [16,17]. Among them, the interstitial deletion of 2p11.2-2p12 was confirmed in only three cases by array CGH [16], high-resolution CGH [17], or qPCR [17]. These deletions ranged in size from 7.5 to 11.4 Mb, encompassing the 390 kb duplicated region of our case. The patient with an 11.4 Mb deletion had psychomotor retardation, mild cutaneous syndactylies, pectus carinatum, hyperlordosis, clubfeet, single umbilical artery, and facial abnormalities, such as low-set ears, broad nasal bridge, frontal bossing, and dolichocephaly [16]. In family 3, a son with a 7.5 Mb deletion presented with impairment of physical development, and intellectual disability, language delay including a pronunciation problem, suspected epilepsy, recurrent Wilms tumor, a large head with frontal bossing, a flat face, and low-set abnormally molded ears [17]. His mother with the same deletion had features similar to her son, such as abnormally molded ears, a severe pronunciation problem, and intellectual disability [17].

Two obstacles hampered us to compare the overlapping phenotype and genomic regions between our duplication patient and three patients with deletions encompassing 2p11.2. Firstly, we do not know whether a dosage effect of genes is involved in both deletions and our duplication, and whether increased or decreased gene dosage would give rise to the same phenotype. Secondly, the genomic regions of 7 reported deletion cases are so large that they could contain more than one gene for a specific phenotype such as intellectual disability. In this circumstance, the simple comparison of overlapping regions in two groups of inappropriately different sized CNVs is not meaningful because our duplication is only 390 kb and the smallest deletion among 7 deletion cases is 7.5 Mb containing a large number of genes.

Therefore, we analyzed known and deduced functions of genes within the duplicated region. While the chromosome band 2p11.2 of 7.2 Mb contains many genes, fifteen annotated genes are located in 390 kb duplicated region of our case; *TCF7L1, TGOLN2, RETSAT, ELMOD3, CAPG, SH2D6, LOC100630918, MAT2A, GGCX, VAMP8, VAMP5, RNF181, TMEM150A, C2orf68,* and *USP39* (Figure 3A, Table 2)*.* These are all protein-coding genes, except the non-coding RNA *LOC100630918*.

The multisystemic disorder in our patient suggests that it is caused by more than one gene or by a single gene encoding a transcription factor regulating various target genes. Among the genes in the duplicated region, *TCF7L1* (transcription factor 7-like 1, formerly named *TCF3*, MIM 604652) is a mediator of the WNT signaling pathway [18]. This gene encodes a transcription factor, which is a member of the T-cell factor/lymphoid enhancer factor. Importantly, *TCF7L1* physically interacts with *CTNNB1* [catenin (cadherin-associated protein), beta 1, MIM 116806] [19], mutations of which cause severe intellectual disability with absent or very limited speech, microcephaly, and spasticity [20]. Since proteins accomplish most of their functions by interacting with other molecules, we wondered whether *TCF7L1* might be involved in intellectual disability by increasing gene dosage from the duplication. Theoretically, *TCF7L1* at the distal duplication junction would express one intact and one truncated transcript, respectively (Figure 4). The truncated one with 3′ end is most likely not expressed due to the lack of the promotor and putative start codon. This was confirmed by qRT-PCR with two different primer pairs—#1 and #2 designed from the 5′ end and 3′ end of this gene (Figures 3A and 4, Table 1), which failed to demonstrate any change in expression in the patient, her healthy mother, and sister (data not shown). This finding makes it highly unlikely that *TCF7L1* is a candidate for intellectual disability.

The gene *USP39* (ubiquitin specific peptidase 39, MIM 611594) is also a good candidate for intellectual disability since alterations of the ubiquitin/proteasome system (UPS) may engender neuronal dysfunction leading to neurological disease and intellectual disability [21]. This gene truncated at the proximal duplication junction would consequently be predicted to produce one intact and one truncated transcript with the 5′ end (Figure 4). Two primer pairs #4 and #5 were designed from the 5′ end and 3′ end of this gene, respectively, for qRT-PCR (Figure 4 and Table 1). The amount of transcript (from two different regions) was not different in the patient, her unaffected mother, and sister (data not shown), suggesting that this gene is not affected by the duplication.

Since the transcription of two truncated genes, *TCF7L1* and *USP39*, is in the same direction, there is a possibility of producing a fusion gene (Figure 4). Practically, it is unlikely that the fusion gene is expressed. If it had been expressed, the amplicon from primer pair #2 would have shown overexpression due to additional expression from the second half of the fusion gene comprising of partial *TCF7L1* with the 3′ end (Figure 4). This hypothesis that the fusion gene was not expressed is confirmed because RT-PCR failed to demonstrate the presence of the putative fusion gene *USP39/TCF7L1* (data not shown), using a forward primer from *USP39* and a reverse primer from *TCF7L1*, (Figure 4 and Table 1).

After eliminating *TCF7L1* and *USP39,* two partially duplicated but unaffected genes, *VAMP8* and *RNF181* become plausible candidate genes for intellectual disability seen in our patient among the 13 genes duplicated at 2p11.2. *VAMP8* (vesicle-associated membrane protein 8, MIM 603177) encodes a protein involved in the fusion of synaptic vesicles with the presynaptic membrane. It interacts with *YWHAE* (tyrosine 3-monooxygenase/tryptophan 5-monooxygenase activation protein, epsilon polypeptide, MIM 605066) [22]. Heterozygous knock-out mice show neuron migration defects, mild defects in spatial working memory, and possibly enhanced anxiety in an elevated plus-maze test. This phenotype coupled with the genetic association between one SNP in the 5′ flanking region of *YWHAE* and schizophrenia suggested this gene a possible susceptibility gene for this psychiatric disorder [23]. This makes *VAMP8* an attractive candidate gene for intellectual disability. *RNF181* (ring finger protein 181, MIM 612490) has E3 ubiquitin ligase activity [24] and because ubiquitination plays a crucial role in neurodevelopment as aforementioned [21], this is a good candidate for intellectual disability.

At least, five genes, *HUWE1* (MIM 300697), *PARK2* (MIM 602544), *UBE3A* (MIM 601623), *UBE3B* (MIM 608047), *RNF216* (MIM 609948), encoding E3 ubiquitin ligases, have been involved in intellectual disability or another neurological phenotype. Mutations of *HUWE1* (HECT, UBA and WWE domain containing 1) are associated with X-linked intellectual disability [25] and *PARK2* (Parkinson protein 2) is mutated in Parkinson patients [26]. Genetic alterations of *UBE3A* (ubiquitin protein ligase E3A) cause Angelman syndrome characterized by intellectual disability, seizures, frequent smiling and laughter, and abnormal gait [27]. Biallelic loss-of-function mutations of *UBE3B* (ubiquitin protein ligase E3B) cause autosomal recessive blepharophimosis-ptosis-intellectual-disability syndrome [28]. Inactivating mutations of *RNF216* (ring finger protein 216) cause Gordon Holmes syndrome, which is characterized by cerebellar ataxia, dementia, and hypogonadotropic hypogonadism [29].

Additionally, *CUL4B* (cullin 4B, MIM 300304) associated with syndromic X-linked intellectual disability is a scaffold subunit of E3 ubiquitin-protein ligase complex [30] and *UBE2A* (ubiquitin-conjugating enzyme E2A, MIM 312180) encoding E2 ubiquitin-conjugating enzyme are associated with X-linked intellectual disability [31]. An unaffected individual with a 36 kb deletion encompassing whole gene *RNF181* has been reported as aforementioned [13]. However, we still cannot exclude the possibility that overexpression from a duplication, which was confirmed by qRT-PCR (Figure 3F), rather than haploinsufficiency, might cause intellectual disability.

Among the duplicated genes, *CAPG* [capping protein (actin filament), gelsolin-like, MIM 153615] encodes a macrophage-capping protein, which controls actin-based motility in non-muscle cells. Homozygous mutant mice lacking this gene manifest increased susceptibility to some bacterial infections [32]. This gene might also have had some influence on the patients’ phenotype as a result of the duplication, given that qRT-PCR showed its overexpression in our patient with recurrent fungal infection on the skin of her hands (Figures 1I, J, and 3D). The patient commonly placed her hands into her mouth, biting her fingers with self-injurious behavior. Her skin lesions were sometimes complicated with fungal infections and treated with antifungal cream, clotrimazole.

Lastly, two genes, *ELMOD3* and *GGCX*, involved in recessive disorders were ruled out as candidate genes because of the absence of associated clinical features of these two genes in our patient with a likely autosomal dominant phenotype due to the heterozygous duplication. A homozygous missense mutation of *ELMOD3* (ELMO/CED-12 domain containing 3, MIM 615427) was found by whole exome sequencing combined with homozygosity mapping in a Pakistani family with autosomal recessive nonsyndromic deafness-88 (DFNB88, MIM 615429) [33], while homozygous missense and compound heterozygous mutations of *GGCX* (gamma-glutamyl carboxylase, MIM 137167) cause vitamin K-dependent coagulation defect (MIM 277450) [34,35] and pseudoxanthoma elasticum-like Disorder with multiple coagulation factor deficiency (MIM 610842) [36].

## Conclusions

In summary, here we report the first interstitial duplication of 2p11.2 associated with syndromic intellectual disability. Based on the functional characterization and qRT-PCR of the genes, we suggest three positional candidate genes—*VAMP8* and *RNF181* for intellectual disability and *CAPG* for recurrent infection. In concert with the qRT-PCR data, we propose increased gene dosage as the underlying mechanism of the two phenotypes.

## Methods

For high resolution karyotype, peripheral blood lymphocytes from the patient, mother, and younger sister were cultured with Phytohematoagglutinin and harvested for cytogenetic analysis using standard techniques. Chromosome analysis was performed on GTL-banded chromosomes at an approximately 550 band level.

Methylation studies of the 15q11-q13 region was performed with enzymes *Hind*III and *Cfo*I and Southern blot analysis with probe *PW71B*.

DNA copy number variants were investigated using a 400 k whole genome oligonucleotide array (GPL9777) employing the protocols for array CGH provided by the manufacturer (Agilent, Santa Clara, USA). Image analysis, normalization, and annotation were done with Feature Extraction 10.5.1.1 (Agilent, Santa Clara, USA) using the default settings. Data visualization and further analysis was performed with GenomeCAT (http://www.molgen.mpg.de/~abt_rop/molecular_cytogenetics/CGHPRO.html). CNVs were determined by circular binary segmentation [37].

To confirm the duplicated region, three primer pairs were designed for real-time quantitative PCR (qPCR)—pairs #1 and #2 from the 5′ and 3′ ends of the gene *TCF7L1*, respectively, and #3 from the gene *TMEM150A* (Figure 3A, Table 1). Primer pair #1 was designed from the outside of the duplicated region as a negative control, whereas primer pairs #2 and #3 were designed from within the duplicated region (Figure 3A). qPCR was performed using an ABI 7300 Realtime PCR system (Applied Biosystem, Foster City, CA, USA) according to the manufacturer’s instructions (Figure 3C). The copy number was measured relative to *GAPDH*.

For real-time reverse transcriptase quantitative PCR (qRT-PCR), RNA was extracted from lymphoblastoid cell lines by Trizol Reagent (Invitrogen, Calsbad, CA, USA). Total RNA was reverse-transcribed into cDNA by RevertAid First-strand cDNA synthesis (Thermo Fisher Scientific, Glen Burnie, MA, USA). qRT-PCR for *TCF7L1*, *CAPG*, *VAMP8*, *RNF181*, and *USP39* was performed with SYBR (RT2 SYBR Green, Qiagen, Gaithersburg, MD, USA) on an ABI 7300 Realtime PCR system (Applied Biosystem, Foster City, CA, USA) according to the manufacturer’s instructions. For three genes, *TCF7L1*, *CAPG*, and *USP39*, two primer pairs were designed per gene, whereas one primer pair was designed for two genes, *VAMP8* and *RNF181* (Table 1).

To amplify the putative fusion gene *USP39/TCF7L1*, reverse transcriptase PCR (RT-PCR) was performed with a forward primer from *USP39* and a reverse primer from *TCF7L1*, respectively. The forward primer was designed from exon 6 of *USP39* because the breakpoint was located between intron 7 (minimum breakpoint) and intron 10 (maximum breakpoint), and an exon 7 fragment is too small to analyze. The reverse primer was designed complementary to exon 5 of *TCF7L1*, since the breakpoint lies in intron 3 and exon 4 is too short (Figure 4, Table 1).

To better define the clinical phenotype associated with copy number variation of 2p11.2, we compared this case with previously reported interstitial deletions encompassing 2p11.2 and assigned individual phenotypes to individual genes in the duplicated region.

Ethical approval was granted for this study by an interdisciplinary institutional reviewer board of Georgia Regents University.

### Consent

Written informed consent was obtained from the patient’s mother for the publication of this report and any accompanying images.

## Abbreviations

aCGH: Array comparative genomic hybridization; EEG: Electroencephalography; FISH: Fluorescent *in situ* hybridization; qPCR: Real-time quantitative PCR; qRT-PCR: Real-time reverse transcriptase quantitative PCR.

## Competing interests

The authors declare that they no competing interest.

## Authors’ contributions

KRJ drafted the manuscript with HGK. HGK supervised the design of the study and edited the manuscript. RU performed microarray. SK and LCL interpreted the results and reviewed the manuscript with many constructive suggestions. All authors have read and approved the final manuscript.
